# Kognitiv-motivationale Schüler*innenprofile und ihre Bedeutung für die Schüler*innenwahrnehmung der Lernunterstützung durch die Lehrperson

**DOI:** 10.1007/s42010-021-00100-3

**Published:** 2021-03-10

**Authors:** Merle Ruelmann, Loredana Torchetti, Sandra Zulliger, Alois Buholzer, Anna-Katharina Praetorius

**Affiliations:** 1 Pädagogische Hochschule Luzern, Luzern, Schweiz; 2 Pädagogische Hochschule Bern, Bern, Schweiz; 3 Universität Zürich, Zürich, Schweiz

**Keywords:** Schülerprofile, Latente Profilanalyse, Kognitiv-motivationale Voraussetzungen, Wahrnehmung, Lernunterstützung, Student Profiles, Latent Profile Analysis, Cognitive-motivational Characteristics, Student Perception, Learning Support

## Abstract

Es ist eine zentrale Aufgabe von Lehrpersonen, Lernende während des Unterrichts individuell zu unterstützen. Im Hinblick auf die Nutzung von Unterstützungsangeboten spielt es eine entscheidende Rolle, wie diese Unterstützung von Schüler*innen wahrgenommen wird. Empirische Studien zeigen, dass die Wahrnehmung des Unterrichts interindividuell stark variiert. Die Ergebnisse deuten darauf hin, dass diese Variabilität auf unterschiedliche kognitive und affektiv-motivationale Voraussetzungen von Schüler*innen zurückgeführt werden kann. Bisher liegen jedoch kaum differenzielle Untersuchungen zur Schüler*innenwahrnehmung der Lernunterstützung vor, die systematisch Zusammenhänge mit individuellen Voraussetzungen in den Blick nehmen. An dieser Stelle setzt die vorliegende Studie mit dem Ziel an, mittels eines personenzentrierten Ansatzes zu einem besseren Verständnis unterschiedlicher Bedürfnisse hinsichtlich der Lernunterstützung beizutragen. Mittels latenter Profilanalysen wurden die Daten von 633 Mathematikschüler*innen der vierten Jahrgangsstufe ausgewertet. Auf diese Weise konnten vier verschiedene Schüler*innenprofile identifiziert werden: (1) starkes Profil, mit hoch ausgeprägter Selbstwirksamkeit, hoch ausgeprägter intrinsischer Motivation und hoch ausgeprägtem Vorwissen in Mathematik; (2) motiviertes Profil mit hoch ausgeprägter intrinsischer Motivation, durchschnittlicher Selbstwirksamkeit und geringem Vorwissen; (3) unmotiviertes Profil mit geringer Selbstwirksamkeit, geringer intrinsischer Motivation und mittlerem Vorwissen; (4) überfordertes Profil mit niedrigen Werten aller Variablen. Lernende mit starken oder motivierten Profilen nahmen die Lernunterstützung ihrer Lehrperson positiver wahr als Schüler*innen mit überforderten und unmotivierten Profilen. Diese Ergebnisse zeigen deutliche Unterschiede in der individuellen Wahrnehmung von Lernunterstützung und weisen diesbezüglich auf weiteren Forschungsbedarf hin.

## Abstract (en)

Providing adaptive learning support during instruction is a pivotal task for teachers. Nevertheless, the students’ perceptions play a major role in how students can benefit from this support. Research has shown that these perceptions of instruction vary widely across learners and that such differences in students’ perception can be explained by their cognitive and motivational characteristics. To date, however, there is only limited systematic research on differences in students’ perception of teachers’ learning support and on how these differences relate to different configurations of students’ characteristics. This study aims to shed light on these aspects by using a person-centered approach, and intends to contribute to an improved understanding of student needs regarding learning support. By conducting latent profile analyses on student data from 633 fourth-grade mathematics students, we identified four distinct student profiles: (1) strong profile with high self-efficacy, high intrinsic motivation, and a high level of prior knowledge in mathematics; (2) motivated profile with high intrinsic motivation, medium self-efficacy, and a low level of prior knowledge; (3) unmotivated profile with low self-efficacy and intrinsic motivation, but a medium level of prior knowledge; and (4) struggling profile with low values on all variables. Students with strong and interested profiles perceived their teachers’ learning support more positively than students with struggling and unmotivated profiles. These results point to major differences between individual perceptions of learning support, and highlight the need for further research.

## Einleitung

Lehrpersonen haben die anspruchsvolle Aufgabe, die heterogenen Lernbedürfnisse ihrer Schüler*innen im Unterricht adäquat zu adressieren. Eine formativ ausgerichtete individuelle Lernunterstützung von Schüler*innen ist eine zentrale Möglichkeit, dies gezielt umzusetzen (Krammer 2016; Schnebel 2017). Schlüsselkomponenten einer solchen Lernunterstützung sind die lernbegleitende Diagnose des Lernstands sowie informatives Feedback, das an diese Diagnose anknüpft (Buholzer et al. 2020). Diesem Ansatz wird ein hohes Potenzial für die Förderung des Lernfortschritts zugeschrieben (z. B. Black und Wiliam 1998; Chin 2006; Hattie 2009; Ruiz-Primo und Furtak 2006). Jedoch muss gemäß dem Angebots-Nutzungs-Modell angenommen werden, dass ihr Erfolg wesentlich von der Wahrnehmung und Nutzung durch die Schüler*innen abhängt (Helmke 2014; Vieluf et al. 2020).

Tatsächlich zeigen Ergebnisse der Unterrichtsqualitätsforschung, dass Schüler*innen derselben Klassen die Lernunterstützung interindividuell deutlich unterschiedlich wahrnehmen (Corbin et al. 2020; den Brok et al. 2006; Downer et al. 2015; Göllner et al. 2018; Rakoczy et al. 2008). Diesen Divergenzen genauer auf den Grund zu gehen, kann dazu beitragen, Unterstützungsbedürfnisse von Lernenden besser zu verstehen und Schlüsse für eine adaptive Gestaltung von Lernunterstützung zu ziehen. Insbesondere in der Primarstufe erscheint dies relevant, da hier nicht nur die Heterogenität zwischen Schüler*innen besonders hoch ausgeprägt ist, sondern darüber hinaus jüngere Schüler*innen stärker als ältere auf die Lernunterstützung der Lehrperson angewiesen sind (Zee und de Bree 2017).

Erste empirische Befunde zur Erklärung solcher Divergenzen in der Unterrichtswahrnehmung deuten darauf hin, dass diese in Verbindung mit kognitiven (z. B. Vorwissen) und affektiv-motivationalen (z. B. Selbstwirksamkeit) Lernvoraussetzungen von Schüler*innen stehen (Clausen 2002; Ditton 2002; Göllner et al. 2018; Igler et al. 2019). Zugleich wird theoretisch angenommen, dass das reziproke Wechselspiel kognitiver und affektiv-motivationaler Lernvoraussetzungen von Schüler*innen für die Wahrnehmung und Nutzung von Unterrichtsangeboten entscheidend ist (Vieluf et al. 2020). Bislang ist jedoch weitgehend ungeklärt, in welchem Zusammenhang konkret eine solche Kombination kognitiver und affektiv-motivationaler Voraussetzungen mit der Wahrnehmung von Lernunterstützung steht, insbesondere dann, wenn kognitive und affektiv-motivationale Merkmale unterschiedliche Ausprägungen aufweisen (z. B. hoch ausgeprägtes Vorwissen gepaart mit geringer Selbstwirksamkeit).

An dieser Stelle setzt die vorliegende Studie an. Im Zentrum steht die Frage, ob zwischen Primarschüler*innen Unterschiede in der Wahrnehmung der Lernunterstützung durch ihre Lehrperson bestehen und wie sich etwaige Unterschiede erklären lassen. Mittels eines personenzentrierten Vorgehens werden anhand des Vorwissens der intrinsischen Motivation und Selbstwirksamkeit in Mathematik kognitiv-motivationale Schüler*innenprofile identifiziert. Personenzentrierte Ansätze eigenen sich sehr gut, um Schüler*innengruppen mit unterschiedlichen Merkmalskonfigurationen abzubilden, da mehrere Lernvoraussetzungen simultan untersucht und Wahrnehmungsdivergenzen zwischen den Gruppen differenziell beleuchtet werden können.

Zur theoretisch-konzeptuellen Grundlegung wird im Folgenden zuerst das Konstrukt der individuellen Lernunterstützung erläutert (Abschn. 2.1). Danach werden interindividuelle Wahrnehmungsunterschiede thematisiert (Abschn. 2.2), und es wird auf die diesbezügliche Bedeutung individueller Voraussetzungen eingegangen (Abschn. 2.3). Des Weiteren werden Vorteile eines personenzentrierten Untersuchungsansatzes aufgezeigt (Abschn. 2.4), woraus schließlich die Fragestellungen und die Hypothesen der vorliegenden Studie abgeleitet werden (Abschn. 2.5).

## Theorie und Forschungsstand

### Individuelle Lernunterstützung in Unterrichtsinteraktionen

Die Unterstützung des Lernens kann einerseits auf der Makroebene erfolgen, beispielsweise bei der Planung von Unterricht und der Auswahl geeigneter, differenzierter Aufgaben. Andererseits kann sie in der direkten Unterrichtsinteraktion zwischen Lehrperson und einzelnen Schüler*innen lernbegleitend stattfinden. Diese Unterstützung im Dialog wird als „individuelle Lernunterstützung“ bezeichnet (Krammer 2016). Sie erlaubt es Lehrpersonen, Adaptionen auf der Mikroebene vorzunehmen und zwischen dem Lernangebot und dessen Nutzung durch die Schüler*innen zu vermitteln (Schnebel 2017). Der individuellen Lernunterstützung kommt deshalb ein großes Potenzial für die Förderung von Lernprozessen zu. Entsprechend gilt sie als wichtiges Merkmal eines adaptiven Unterrichts sowie der Unterrichtsqualität (Krammer 2016; Pauli und Reusser 2015). Wie Lehrpersonen Schüler*innen unterrichtsbegleitend individuell unterstützen können, wurde in der Literatur vielfältig beschrieben und empirisch untersucht (für eine Übersicht: Siemon et al. 2018). Im englischsprachigen Raum wurden Unterstützungshandlungen primär unter den Begriffen „scaffolding“ (z. B. van de Pol et al. 2010) und „formative assessment“ (z. B. Black und Wiliam, 1998) in den Blick genommen. In verschiedenen Arbeiten werden wiederkehrend zwei Schlüsselkomponenten der individuellen Lernunterstützung genannt: die lernbegleitende Diagnose und das lernförderliche Feedback (Black und Wiliam, 1998; Buholzer et al. 2020; Chin 2006; Hattie und Timperley 2007; Jurik et al. 2014; Wullschleger 2017), welche in einem interdependenten Zusammenhang stehen. Bei der lernbegleitenden Diagnose erheben Lehrpersonen, z. B. durch anregende Fragen, fortlaufend Informationen, die Einblicke in das Denken und den Lernstand der Lernenden geben. Effektives Feedback baut auf den dadurch gewonnenen Einsichten auf und fördert den individuellen Lernprozess mit gezielten Informationen (Shute 2008). In der Forschung zum formativen Assessment konnte gezeigt werden, dass diesen beiden Unterstützungshandlungen in beträchtlichem Maße zur Förderung des Lernfortschritts beitragen können (Black und Wiliam, 1998; Chin 2006; Hattie 2009; Hattie und Timperley 2007; Jurik et al. 2014; Ruiz-Primo und Furtak 2006; Shute 2008).

### Interindividuelle Wahrnehmungsunterschiede

Schüler*innen sind die Adressat*innen des Unterrichts, daher ist es von hoher Relevanz, wie sie diesen und damit auch die Lernunterstützung wahrnehmen (Clausen 2002; Ditton 2002). Das im deutschsprachigen Raum weit verbreitete Angebots-Nutzungs-Modell geht davon aus, dass Lernende aktive (Ko‑)Konstrukteur*innen ihres eigenen Lernfortschrittes sind. Die Wirkung der Lernunterstützung hängt somit nicht allein vom Angebot der Lehrperson ab, sondern auch von dessen Wahrnehmung und Nutzung durch die Schüler*innen (Helmke 2014). Wahrnehmung und Nutzung sind individuelle Prozesse, die vom subjektiven Erleben einzelner und vom weiteren Kontext geprägt sind. Das Angebot hat infolgedessen nicht auf alle Schüler*innen die gleiche Wirkung (Vieluf et al. 2020).

Aktuelle Forschungsbefunde bestätigen eine große Varianz in der Unterrichtswahrnehmung, die zu 70–90 % auf interindividuelle Wahrnehmungsunterschiede zwischen Schüler*innen innerhalb derselben Klassen zurückgeführt werden kann (Fauth et al. 2014). Dies bedeutet, dass Schüler*innen selbst unter gleichen Unterrichtsbedingungen zu divergenten Einschätzungen des Unterrichts und der Lernunterstützung gelangen (Corbin et al. 2020; den Brok et al. 2006; Downer et al. 2015; Igler et al. 2019; Göllner et al. 2018; Rakoczy et al. 2008). Die individuelle Wahrnehmung der Lernunterstützung ist des Weiteren bedeutungsvoll für den Lernfortschritt. Es liegen empirische Befunde vor, wonach eine individuell positive Einschätzung der Lernunterstützung die Anstrengungsbereitschaft (Schenke 2018), die Motivation (Dietrich et al. 2015; Fast et al. 2010) und die Leistung (Harks et al. 2014; Rakoczy et al. 2013) verbessern kann.

Bisherige Studien zu Wahrnehmungsunterschieden fokussierten vornehmlich auf den Bereich der Sekundarschule. In der Primarschule kommt der Lernunterstützung jedoch eine besondere Relevanz zu, weil Primarschüler*innen stärker als ältere Schüler*innen auf die Ko-Regulierung ihrer lernbezogenen Verhaltensweisen angewiesen sind (Zee und de Bree 2017). Gleichzeitig ist die Heterogenität in der Primarschule besonders hoch ausgeprägt. Auch zeichnet sich u. a. für das Fach Mathematik Forschungsbedarf ab, da die Motivation in Mathematik im Laufe der Primarschulzeit abnimmt (Gottfried et al. 2007; Spinath und Steinmayr 2008). Des Weiteren wurde die individuelle Lernunterstützung in den genannten Studien unterschiedlich operationalisiert. Entweder wurde sie als ein Teilaspekt von Unterrichtsqualität mittels einer globalen Skala erfasst, die nicht zwischen verschiedenen Komponenten der Lernunterstützung unterschied, oder es wurde ausschließlich das Feedback untersucht (Harks et al. 2014; Rakoczy et al. 2013). Eine differenzierte Betrachtung der Lernunterstützung aus Schüler*innensicht steht bislang aus, obwohl sie weiterführende Informationen für die adaptive Gestaltung der Lernunterstützung liefern könnte.

### Die Bedeutung individueller Voraussetzungen für Wahrnehmungsunterschiede

Im Angebots-Nutzungs-Modell wird individuellen Lernvoraussetzungen eine zentrale Bedeutung für die Wahrnehmung und die Nutzung des Lernangebots beigemessen (Helmke 2014). Unterschiede in der individuellen Wahrnehmung können u. a. durch Mechanismen der Informationsverarbeitung erklärt werden. Diese erfolgt auf der Basis von kognitiven und affektiv-motivationalen Voraussetzungen, die wie ein Filter auf die persönliche Wahrnehmung wirken und so zu Divergenzen im schülerseitigen Erleben von Lernunterstützung führen können (Bless et al. 2004; Ilgen et al. 1979).

Bislang ist hinsichtlich der Lernunterstützung noch nicht geklärt, welche kognitiven und affektiv-motivationalen Voraussetzungen von Lernenden für die Prozesse der Wahrnehmung und der Nutzung von Bedeutung sind. Eine entscheidende kognitive Voraussetzung dürfte jedoch das domänenspezifische *Vorwissen* darstellen. Es hängt vom Vorwissen ab, wie Lernende neue Informationen aufnehmen, da diese in ein bereits vorhandenes Wissensnetz integriert werden müssen (Gruber und Stamouli 2015). Eine effektive Lernunterstützung sollte daher u. a. am Vorwissen von Lernenden ansetzen (Wullschleger 2017).

Was die affektiv-motivationalen Voraussetzungen anbelangt, so wird die Lernmotivation von Schüler*innen in der modernen Motivationspsychologie als komplexes Zusammenspiel internaler Merkmale und Prozesse beschrieben, welche Intentionen und Handlungen steuern (Pintrich 2003). Gemäß der Erwartungs-Wert-Theorie können leistungsbezogene Verhaltensweisen anhand individueller Überzeugungen zu den eigenen Fähigkeiten (Erfolgserwartungen) sowie durch den affektiven und den kognitiven Wert von Tätigkeiten (Wertkomponenten) erklärt werden (Eccles 2005). *Selbstwirksamkeit* (Erfolgserwartung) und *intrinsische Motivation* (Wertkomponente) sind wichtige Merkmale dieser beiden Dimensionen, sodass die Lernmotivation exemplarisch anhand dieser Schlüsselkomponenten modelliert werden kann.

Die wenigen Studien, die Zusammenhänge zwischen kognitiven und motivationalen Merkmalen und der Unterrichtswahrnehmung bislang untersuchten, fokussierten vorwiegend auf die Sekundarschule (Ausnahme: Igler et al. 2019) und analysierten den Einfluss der Variablen auf die Unterrichtswahrnehmung von Schüler*innen separat. Zum Einfluss des *Vorwissens* (bzw. Grundfertigkeiten, Vorleistung, Eingangskompetenzen) auf die Wahrnehmung von Unterricht (z. B. Klassenführung, Lerntechniken, Lehrperson) finden sich Hinweise, wonach Schüler*innen mit geringem Vorwissen den Unterricht negativer wahrnehmen als jene mit großem Vorwissen (Ditton 2002; Göllner et al. 2018; Schiepe-Tiska et al. 2016). Umgekehrt führt ein umfangreiches Vorwissen dazu, dass Aufgaben als weniger schwierig erachtet werden und die Lehrperson als motivierender wahrgenommen wird (Igler et al. 2019). Auf die Lernunterstützung übertragen, könnte dies beispielsweise bedeuten, dass diese von einer Schülerin mit viel Vorwissen positiv eingeschätzt wird, weil sie neue Informationen schnell einordnen kann und deshalb einen geringen Unterstützungsbedarf aufweisen dürfte. Ein Schüler mit wenig Vorwissen könnte hingegen zu einer negativeren Einschätzung gelangen, da er möglicherweise mehr Unterstützungsbedarf hat. Befunde von Schiepe-Tiska et al. (2016) deuten auf einen entsprechenden positiven Zusammenhang zwischen hoher Mathematikkompetenz und der wahrgenommenen Lernunterstützung hin, während dieser Zusammenhang bei Göllner et al. (2018) nicht gefunden wurde.

Zum Einfluss von *Selbstwirksamkeit* (bzw. Selbstkonzept) liegen ebenfalls widersprüchliche Befunde vor. So fanden Jurik et al. (2015) einen positiven Zusammenhang zwischen dem Selbstkonzept und der Unterrichtseinschätzung in Deutsch, jedoch einen entgegengesetzten Zusammenhang für Mathematik, während Schiepe-Tiska et al. (2016) für die wahrgenommene Lernunterstützung gar keinen Zusammenhang mit der Selbstwirksamkeit nachweisen konnten. Zum Einfluss der *intrinsischen Motivation* wiederum finden sich Hinweise darauf, dass Schüler*innen mit Freude und Interesse am Mathematikunterricht (bzw. an der Schule; Igler et al. 2019) ihre Lehrperson und die Unterstützung positiver wahrnehmen als weniger intrinsisch motivierte (Ditton 2002; Jurik et al. 2015; Schiepe-Tiska et al. 2016).

Insgesamt deuten die Befunde drauf hin, dass Vorwissen, Selbstwirksamkeit und intrinsische Motivation zentrale Faktoren für die Wahrnehmung der Lernunterstützung sind und hohe Ausprägungen dieser Merkmale tendenziell mit einer positiveren Unterrichtswahrnehmung einhergehen. Die vorliegenden Befunde sind jedoch, wie bereits festgehalten, teilweise widersprüchlich und beziehen sich überwiegend nicht explizit auf die individuelle Lernunterstützung durch die Lehrperson. Des Weiteren wurden die Merkmale einzeln untersucht, obwohl Wahrnehmung und Nutzung von Unterrichtsangeboten als Prozesse der reziproken Wechselwirkung kognitiver und affektiv-motivationaler Merkmale beschrieben werden (Vieluf et al. 2020).

Aktuelle Studien bestätigen, dass die Kombination von Schüler*innenmerkmalen für das Lernen von Bedeutung ist (Corpus und Wormington 2014; Jurik et al. 2014; Lazarides et al. 2018, 2019; Linnenbrink-Garcia et al. 2012; Seidel 2006; Seidel et al. 2016; Südkamp et al. 2018). Zum Beispiel untersuchten Linnenbrink-Garcia et al. (2012) den Einfluss von Interesse, Selbstwirksamkeit und Vorwissen auf Konzeptwechselprozesse im Biologieunterricht der neunten Jahrgangsstufe. Sie fanden heraus, dass die Variablen jeweils einzeln betrachtet keine Unterschiede im Lernprozess erklären konnten. Erst mit einem personenzentrierten Ansatz wurden Unterschiede gefunden, die auf vier Schüler*innenprofile mit unterschiedlichen kognitiv-motivationalen Merkmalskombinationen zurückgeführt werden konnten: 1. Schüler*innen mit hoch ausgeprägtem Interesse, hoch ausgeprägter Selbstwirksamkeit und hoch ausgeprägtem Vorwissen, 2. Lernende mit moderat ausgeprägtem Interesse, moderat ausgeprägter Selbstwirksamkeit und geringem Vorwissen, 3. Schüler*innen mit geringem Interesse, geringer Selbstwirksamkeit und hoch ausgeprägtem Vorwissen sowie 4. Schüler*innen mit hoch ausgeprägtem Interesse, hoch ausgeprägter Selbstwirksamkeit und moderat ausgeprägtem Vorwissen.

Es kann angenommen werden, dass diese Kombination von Merkmalen auch für die Wahrnehmung von Lernunterstützung entscheidend ist. In diese Richtung deuten Ergebnisse, die ebenfalls aus dem Bereich der Sekundarstufe stammen. Im Physikunterricht der neunten Jahrgangsstufe untersuchte Seidel (2006) den Zusammenhang zwischen kognitiven und affektiv-motivationalen Voraussetzungen von Schüler*innen und der Unterrichtswahrnehmung personenzentriert. Anhand der allgemeinen kognitiven Fähigkeiten, des Vorwissens, des Selbstkonzepts und des Interesses konnte sie fünf verschiedene Schüler*innenprofile identifizieren: stark, uninteressiert, unterschätzend, überschätzend, schwach. Schüler*innen mit einem starken oder überschätzenden Profil beurteilten die Unterrichtsqualität positiver als Lernende mit einem unterschätzenden oder überforderten Profil.

Für den Bereich der Primarstufe liegen bislang erst wenige empirische Untersuchungen zu Schüler*innenprofilen vor. Es existieren nur zwei Studien, welche sowohl kognitive als auch affektiv-motivationale Voraussetzungen personenzentriert untersuchten (Lazarides et al. 2018; Südkamp et al. 2018). In der Studie von Südkamp et al. (2018) wurde untersucht, wie unterschiedliche Voraussetzungen von Schüler*innen von Lehrpersonen wahrgenommen werden. Die Autorinnen unterscheiden zwischen konsistenten und inkonsistenten Schüler*innenprofilen, bei denen kognitive und affektiv-motivationale Voraussetzungen entweder konvergent oder divergent ausgeprägt sind. Bei konsistenten Profilen weisen kognitive und motivationale Merkmale gleiche Merkmalsausprägungen auf (hoch, mittel oder niedrig). Inkonsistente Profile umfassen hingegen Schüler*innen, deren kognitive und motivationale Voraussetzungen voneinander abweichen. Südkamp et al. (2018) fanden ein inkonsistentes Profil mit durchschnittlich ausgeprägten kognitiven Fähigkeiten und niedrig ausgeprägten motivationalen Variablen, das von Lehrpersonen nicht akkurat erkannt wurde. In Studien im Bereich der Sekundarstufe wurden sogar mehrere inkonsistente Schüler*innenprofile identifiziert (z. B. Seidel 2006; Linnenbrink-Garcia et al. 2012), was darauf hindeutet, dass Schüler*innenprofile in der Primarstufe möglicherweise weniger differenziert sind. In der Studie von Lazarides et al. (2018) wurde die motivationale Entwicklung im Mathematikunterricht über die Primarschulzeit hinweg personenzentriert untersucht. Die Befunde zeigen, dass nicht alle Schüler*innen gleichermaßen von negativen motivationalen Entwicklungstendenzen betroffen waren, sondern Lernende mit inkonsistenten Profilen eine besonders gefährdete Gruppe darstellten (Lazarides et al. 2018).

Zusammenfassend dürfte es sich angesichts der bislang vorliegenden Befunde als aufschlussreich erweisen, insbesondere die Wahrnehmung der Lernunterstützung von Lernenden mit inkonsistenten Profilen genauer zu untersuchen. Es ist zu vermuten, dass Lehrpersonen ihr Unterstützungshandeln stärker an der Leistungsfähigkeit von Schüler*innen ausrichten als an motivationalen Merkmalen, da letztere schwieriger fassbar sind und von Lehrpersonen weniger akkurat eingeschätzt werden (Praetorius und Südkamp 2019). Schüler*innen mit inkonsistenten Profilen, insbesondere solche, die zwar über ein hoch ausgeprägtes Vorwissen verfügen, aber gleichzeitig wenig motiviert sind, könnten daher in geringerem Masse im Blickfeld von Lehrpersonen stehen als Schüler*innen mit geringem Vorwissen. Eine Untersuchung der Wahrnehmung der Lernunterstützung von Lernenden mit inkonsistenten Profilen kann Aufschluss über ihre individuellen Bedürfnisse geben und Lehrpersonen in der Folge dabei helfen, diese Bedürfnisse gezielter zu adressieren.

### Vorteile personenzentrierter Analysen

Wie bereits dargelegt, ist es im Hinblick auf Unterschiede in der Wahrnehmung von Lernunterstützung aufschlussreich, kognitive und affektiv-motivationale Voraussetzungen in Kombination in den Blick zu nehmen. Die Kombination von Schüler*innenmerkmalen kann personen- oder variablenzentriert untersucht werden. Im variablenzentrierten Vorgehen werden Interaktionen zwischen zwei oder mehreren Variablen mittels multipler Regressionen modelliert. Auf diese Weise wird der Einfluss von festgelegten Variablenkombinationen im Hinblick auf eine abhängige Variable (im vorliegenden Fall die Lernunterstützung) über die Stichprobe hinweg analysiert. Im Vorfeld muss genau festgelegt werden, welche Interaktionen untersucht sowie in welcher Hierarchie die Interaktionen berücksichtigt werden sollen (Bauer und Shanahan 2007). Insbesondere bei der Analyse mehrerer und komplexer Interaktionen ist dieser Ansatz jedoch limitiert und führt zu Multikollinearitätsproblemen (Pastor et al. 2007). Ein personenzentriertes Vorgehen, wie die latente Profilanalyse, ist deshalb geeigneter, um mehrere kognitive und motivationale Voraussetzungen von Schüler*innen kombiniert in den Blick zu nehmen, da mögliche Interaktionen zwischen den Variablen simultan exploriert werden können. Auf diese Weise lassen sich mittels Wahrscheinlichkeitsschätzungen anhand der kognitiven und der motivationalen Merkmale unabhängig von der Outcomevariable Subgruppen (Schüler*innenprofile) innerhalb der Stichprobe identifizieren, die sich in ihren Merkmalskombinationen unterscheiden. So können einerseits Aussagen über die prozentuale Verteilung der Schüler*innen auf die Profile getroffen werden (z. B. Pastor et al. 2007). Andererseits lässt sich der Zusammenhang mit der abhängigen Variable für die Subgruppen (nicht über die Stichprobe hinweg) in einem separaten Rechenschritt differenziell untersuchen.

### Fragestellungen und Hypothesen

Die vorangegangenen Ausführungen zeigen, dass Unterschiede in der Wahrnehmung von Lernunterstützung bisher kaum personenzentriert untersucht wurden, dieser Aspekt jedoch höchst relevant für ein besseres Verständnis der Verarbeitung und Nutzung von Lernunterstützung ist. Erste Ergebnisse deuten in die Richtung, dass individuelle Merkmalskonfigurationen in Zusammenhang mit der Unterrichtswahrnehmung von Schüler*innen stehen (Seidel 2006). Diesbezüglich besteht u. a. für den Mathematikunterricht in der Primarstufe jedoch nach wie vor Forschungsbedarf, da jüngere Schüler*innen besonders auf die Lernunterstützung durch die Lehrperson angewiesen sind (Zee und de Bree 2017). Gleichzeitig wurden ungünstige motivationale Entwicklungstendenzen in Mathematik nachgewiesen (Lazarides et al. 2018), denen auf der Grundlage eines besseren Verständnisses der Lernunterstützung aus Schüler*innensicht entgegengewirkt werden könnte. Zur Klärung dieser Forschungsdesiderata wurde in der vorliegenden Studie in einem ersten Schritt die folgende Frage untersucht:

#### F1

Lassen sich anhand der Variablen „Vorwissen“, „Selbstwirksamkeit“ und „intrinsische Motivation“ im Mathematikunterricht der Primarschule kognitiv-motivationalen Schüler*innenprofile identifizieren und, wenn ja, welche?

In bisherigen personenzentrierten Studien wurden Schüler*innenprofile anhand einer Vielzahl unterschiedlicher kognitiver und affektiv-motivationaler Merkmale untersucht, wobei Lernende mit inkonsistenten und konsistenten Profilen identifiziert werden konnten (Corpus und Wormington 2014; Lazarides et al. 2018; Linnenbrink-Garcia et al. 2012; Seidel 2006; Südkamp et al. 2018). Die kognitiv-motivationalen Schüler*innenprofile, die sich in den wenigen Studien zur Primarstufe finden ließen (Lazarides et al. 2018; Südkamp et al. 2018), erwiesen sich als weniger differenziert als Profile an weiterführenden Schulen. Es lässt sich daher annehmen, dass sich auch im Mathematikunterricht der Primarstufe anhand der Variablen *Vorwissen, Selbstwirksamkeit* und *intrinsische Motivation* konsistente und inkonsistente Profile finden lassen. Spezifischer wird bezugnehmend auf Südkamp et al. (2018) angenommen, dass sich zumindest zwei konsistente und ein inkonsistentes Profil identifizieren lassen (H1).

Ausgehend von der ersten Forschungsfrage wurde folgende zweite Forschungsfrage untersucht:

#### F2

Wie nehmen Primarschüler*innen mit unterschiedlichen Profilen die Lernunterstützung ihrer Lehrpersonen wahr?

Erste Ergebnisse aus der Sekundarstufe verweisen auf Wahrnehmungsunterschiede in Bezug auf unterschiedliche unterrichtliche Merkmale zwischen Lernenden mit unterschiedlichen Profilen (Lazarides et al. 2019; Seidel 2006). Vor diesem Hintergrund dürfte auch von profilabhängigen Unterschieden in der Wahrnehmung der Lernunterstützung auszugehen sein. Konkret lässt sich unter Bezug auf variablenzentrierte Studien annehmen, dass konsistent hoch ausgeprägte Voraussetzungen tendenziell zu einer positiveren und konsistent niedrige Ausprägungen tendenziell zu einer negativeren Wahrnehmung der Lernunterstützung führen dürften (H2.1). Bezugnehmend auf Seidel (2006) gehen wir außerdem davon aus, dass Schüler*innen mit inkonsistenten Profilen mit einem hoch ausgeprägten Vorwissen, aber gering ausgeprägten motivationalen Voraussetzungen zu einer negativeren Wahrnehmung gelangen als Lernende mit konsistent hoch ausgeprägten Merkmalen (H2.2).

## Methode

### Stichprobe und Datenerhebung

Für die vorliegende Untersuchung wurde ein Teil des Datensatzes der Videostudie *TUFA* (**T**eachers’ **U**se of **F**ormative **A**ssessment) ausgewertet, die von Februar bis Juni 2017 durchgeführt wurde.^1^ An der *TUFA*-Studie (Buholzer et al. 2020) nahmen 633 Schüler*innen der vierten Primarschulklassen aus der Zentralschweiz und ihre 52 Lehrpersonen teil. Eltern und Kinder stimmten der freiwilligen Teilnahme an der Studie gemäß den forschungsethischen Richtlinien schriftlich zu. Durchschnittlich waren die Schüler*innen 10,5 Jahre alt (*SD* = 0,49; *Range:* 9–12,6). Die Stichprobe bestand zu 50,6 % aus Mädchen, und 38 % der Lernenden waren mehrsprachig, d. h., sie hatten angegeben, zu Hause vorwiegend eine andere oder eine weitere Sprache als Deutsch zu sprechen (vgl. Tab. 5 im Anhang).

Vor Beginn einer Unterrichtseinheit zur halbschriftlichen Division erhoben die Lehrpersonen mit einem standardisierten Leistungstest das *Vorwissen* der Schüler*innen. Im Anschluss an die Einführungslektion wurden die Lernenden in Abwesenheit der Lehrperson von geschulten Testleiterinnen anhand eines standardisierten Verfahrens zu ihrer Motivation und ihrer Wahrnehmung der Lernunterstützung befragt. Die Fragebogenitems wurden vorgelesen, um eine Konfundierung der Ergebnisse mit der Lesekompetenz auszuschließen.

### Instrumente

Alle verwendeten Skalen wurden im Vorfeld pilotiert und mittels konfirmatorischer Faktorenanalysen auf interne Konsistenz sowie Abgrenzbarkeit voneinander überprüft. Die Skalenkennwerte sind in Tab. 1 aufgeführt.

**Table 1:** 

Skala	Items	*N*	*M *(*SD*)	Minimum	Maximum	Schiefe	Kurtosis	Cronbachs α
Intrinsische Motivation	5	602	2,00 (0,68)	0,00	3,00	−0,44	−0,43	0,86
Selbstwirksamkeit	4	598	2,25 (0,59)	0,40	3,00	−0,65	−0,21	0,85
Lernunterstützung global	4	608	2,54 (0,48)	0,25	3,00	−1,80	4,77	0,72
Feedback	3	607	1,89 (0,67)	0,00	3,00	−0,15	−0,47	0,72
Lernbegleitende Diagnose	4	603	1,62 (0,56)	0,00	3,00	0,18	−0,32	0,72
Testaufgaben: Vorwissen	36	621	25,24 (7,88)	2,00	36,00	−0,74	−0,18	0,93

*N* Anzahl der Schüler*innen, *M* Mittelwert, *SD* Standardabweichung

#### Skalen zur Motivation: Intrinsische Motivation und Selbstwirksamkeit

Basierend auf der Erwartungs-Wert-Theorie (Eccles 2005) wurde als erwartungsbezogene Komponente der Motivation die *Selbstwirksamkeit* (Beispielitem: „Ich kann fast alle Aufgaben in Mathematik schaffen, wenn ich mich anstrenge.“) und als und wertbezogene Komponente die *intrinsische Motivation *in Mathematik (Beispielitem: „Mathematik macht mir Spaß.“) erhoben. Beide Skalen wurden nach den Originalskalen von Rimm-Kaufman et al. (2015) aus dem Englischen übersetzt. Die Schüler*innen gaben ihre Zustimmung anhand einer vierstufigen Likertskala an (0 = „stimmt nicht“ bis 3 = „stimmt“).

#### Skalen zur Lernunterstützung

Die wahrgenommene Lernunterstützung wurde differenziert mit drei Skalen erfasst. Mittels einer nach Bos et al. (2010) adaptierten und für Primarschüler*innen vereinfachten Skala wurde die Wahrnehmung der Lernunterstützung (*Lernunterstützung global*) erfragt (Beispielitem: „Meine Lehrperson hilft mir im Mathematikunterricht, wenn ich Hilfe brauche.“). Zwei weitere Skalen bezogen sich auf die zentralen Unterstützungshandlungen *lernbegleitende Diagnose* (Beispielitem: „Unsere Lehrperson will genau wissen, wie wir etwas gerechnet haben.“) und* Feedback* (Beispielitem: „Meine Lehrperson sagt mir, was ich noch lernen sollte.“). Die Feedbackskala wurde nach Roos und Wandeler (2012) angepasst, die Skala zur lernbegleitenden Diagnose für das *TUFA*-Projekt (Buholzer et al. 2020) neu entwickelt. Die Einschätzungen wurden in vier Abstufungen vorgenommen, wobei die wahrgenommene Qualität der Lernunterstützung mittels Häufigkeitsangaben operationalisiert wurde (0 = „nie“ bis 3 = „immer“).

#### Leistungstest

In Zusammenarbeit mit Mathematikdidaktiker*innen wurde für die *TUFA*-Studie (Buholzer et al. 2020) ein Leistungstest zur Erfassung des *Vorwissens* zum halbschriftlichen Dividieren entwickelt (u. a. Schulz 2015). Die Testaufgaben umfassten Aufgaben zum Operationsverständnis der Division, zu Zahlbeziehungen sowie zur Multiplikation und Division. Wie die Skalen wurde auch der Leistungstest im Vorfeld pilotiert. Als Grundlage der Auswertung diente ein standardisiertes Manual. Die Aufgaben zu Multiplikation, Division und Zahlbeziehungen wurden dichotom beurteilt (0 = „falsch“; 1 = „richtig“), während zur Auswertung des Operationsverständnisses drei Ausprägungen zur Verfügung standen (0 = „falsch“, 0,5 = „teilweise richtig“; 1 = „richtig“). Als Maß für das Vorwissen wurde anschließend ein Summenscore gebildet.

### Statistische Analysen

Die latenten Profilanalysen (F1) wurden mit Mplus 8 berechnet (Muthén und Muthén 2017). Dazu wurden von den konfirmatorischen Faktorenanalysen F‑Scores der Skalen zur Selbstwirksamkeit und zur intrinsischen Motivation in Mathematik extrahiert. Um die Interpretierbarkeit zu erleichtern, gingen diese Skalen sowie der Summenscore des Vorwissens *z*-standardisiert in die latente Profilanalyse ein. Zwei multivariate Ausreißer, ermittelt durch die Mahalanobis-Distanzen, wurden Pastor et al. (2007) folgend ausgeschlossen.

Mittels der latenten Profilanalyse lassen sich auf der Grundlage von Indikatorvariablen in einer Population latente Subgruppen (Profile) identifizieren (Collins und Lanza 2010). Die ermittelten Profile zeichnen sich durch unterschiedliche Kombinationen von Merkmalsausprägungen aus, und zu jedem dieser Profile werden für jede Versuchsperson Zuordnungswahrscheinlichkeiten geschätzt (Berlin et al. 2014). In der vorliegenden Untersuchung wurden Lösungen für ein bis acht Profile berechnet. Die Entscheidung in Bezug darauf, wie viele Profile die Daten am besten abbilden, wurde anhand statistischer Kennwerte (AIC, BIC, aBIC, Entropy) und Tests (Bootstrapped likelihood ratio-test, BLRT; Lo-Mendel-Rubin likelihood ratio test, LMR; Lo-Mendel-Rubin adjusted likelihood ratio test, aLMR) sowie inhaltsbezogener Überlegungen zur Interpretierbarkeit und Sinnhaftigkeit der Profillösungen im Hinblick auf Theorie und Forschungsstand getroffen (vgl. dazu Berlin et al. 2014; Pastor et al. 2007). Die hierarchische Datenstruktur (Schüler*innen in Schulklassen) wurde mit der Option „type = complex“ in Mplus berücksichtigt. Da der BLRT-Test nicht mit dieser Option durchgeführt werden kann, wurden die Profilanalyse zusätzlich auch ohne diese Korrektur vorgenommen.

In einem zweiten Schritt wurde in Anlehnung an Berlin et al. (2014) mittels des in Mplus implementierten BCH-Verfahrens überprüft, ob sich die Indikatoren zwischen den Profilen signifikant voneinander unterscheiden. Das BCH-Verfahren ist robust für schiefe Verteilungen und wird als multiples Gruppenmodell geschätzt, indem Gewichtungen entsprechend den Messfehlern der latenten Variablen berücksichtig werden (Asparouhov und Muthen 2014). Neben dem Overall-Test liefert das Verfahren auch Tests für die einzelnen Gruppenvergleiche.

In einem dritten Schritt wurde analysiert, ob die Profilzugehörigkeit durch das Geschlecht oder die Mehrsprachigkeit vorausgesagt wird. Dazu wurde das in Mplus implementierte R3STEP-Verfahren angewendet. Das R3STEP ist ein dreischrittiges Verfahren, bei dem der Zuordnungsfehler korrigiert und eine multinomiale logistische Regression berechnet wird. Die Wahrscheinlichkeiten für die Profilzugehörigkeit werden durch die Berechnung der Regression nicht verändert (Asparouhov und Muthen 2014; Vermunt 2010). Fehlende Werte in den Prädiktoren wurden mittels multipler Imputationen geschätzt. Zusätzlich zu den nicht standardisierten *b*-Werten wurden partiell standardisierte logistische Regressionskoeffizienten (Menard 2011) sowie Odd-Ratio-Werte (OR) berechnet. Um die Interpretierbarkeit zu erleichtern, wurden die OR schließlich in Wahrscheinlichkeiten umgerechnet, sodass die Verteilung der Schüler*innen anhand der Profilzugehörigkeit je nach Merkmal dargestellt werden konnte.

Zusammenhänge zwischen den Profilen und der Wahrnehmung der Lernunterstützung (F2) wurden ebenfalls mittels des in Mplus implementierten BCH-Verfahrens untersucht. Auch bei diesem Verfahren werden die Wahrscheinlichkeiten für die Profilzugehörigkeit nicht verändert. Um die Interpretierbarkeit zu erleichtern, gingen die Skalen der Lernunterstützung *z*-standardisiert in die Analyse ein. Der hierarchischen Datenstruktur wurde durch die Verwendung der Funktion „type = complex“ Rechnung getragen. Außerdem wurde eine Maximum-Likelihood-Schätzung mit robusten Standardfehlern (MLR) durchgeführt.

## Ergebnisse

### Latente Profilanalyse

Auf der Grundlage der ersten Forschungsfrage wurde untersucht, ob sich anhand der Variablen *Selbstwirksamkeit, intrinsische Motivation* und *Vorwissen *in der Primarschule unterschiedliche Schüler*innenprofile im Fach Mathematik identifizieren lassen (F1). Tiefere AIC- und BIC-Werte weisen auf einen besseren Modellfit hin. Wie in Tab. 2 ersichtlich wird, sinken diese Werte bis zur Schwelle von vier Profilen deutlich, ab fünf Profilen jedoch nur noch geringfügig, sodass eine relative Modellverbesserung bei bis zu vier Profilen vorliegt.

**Table 2:** 

Profile	Loglike	AIC	BIC	Adj. BIC	Entropy	VLMR *p (p*_*complex*_*)*	aLMR *p (p*_*complex*_*)*	BLRT *p*
1	−2606,59	5225,18	5251,86	5232,82	1,00	–	–	–
2	−2450,93	4921,85	4966,33	4934,58	0,73	311,33 0,00 (0,00)	299,71 0,00 (0,00)	311,33 0,00
3	−2400,59	4829,18	4891,44	4846,99	0,80	100,68 0,00 (0,02)	96,92 0,00 (0,02)	100,68 0,00
4	−2371,27	4778,54	4858,59	4801,44	0,75	58,64 0,04 (0,15)	56,45 0,04 (0,16)	58,64 0,00
5	−2355,93	4755,86	4853,70	4783,85	0,77	30,68 0,23 (0,46)	29,54 0,24 (0,47)	30,68 0,00
6	−2332,59	4717,18	4832,812	4750,26	0,80	46,68 0,02 (0,14)	44,93 0,02 (0,15)	46,68 0,00
7	−2304,13	4668,26	4801,68	4706,43	0,83	56,92 0,01 (0,14)	54,80 0,01 (0,15)	*56,92* 0,00
8	−2289,50	4646,99	4798,20	4690,25	0,82	29,27 0,24 (0,57)	28,17 0,26 (0,58)	29,27 0,00

*Loglike* Loglikelihood value, *AIC* Akaike Information Criterion, *BIC* Bayesian Information Criterion, *VLMR* Vuong-Lo-Mendell-Rubin Likelihood Ratio Test, *aLMR* Lo-Mendell-Rubin adjustet Likelihood Ratio Test, *BLRT* Parametric Bootstrapped Likelihood Ratio Test, *p* Signifikanzwert,* p*_*(complex)*_ Signifikanzwert bei Berechnung mit „type = complex“

Hohe Werte in der Entropie (über 0,80) sind sowohl bei drei als auch bei sechs bis acht Profilen gegeben. Die statistischen Tests VLMR und aLMR verweisen auf signifikante Modellverbesserungen bei bis zu drei (mit Berücksichtigung der hierarchischen Datenstruktur mittels „type = complex“) bzw. vier Profilen (ohne Berücksichtigung der hierarchischen Datenstruktur), während der BLRT eine stetige Verbesserung mit zunehmender Profilanzahl anzeigt.

Zusammengefasst lassen die statistischen Kennwerte keine eindeutige optimale Profillösung erkennen. Sie deuten jedoch darauf hin, dass Modelle ab fünf Profilen an die Daten überangepasst sind und eine Profillösung mit drei oder vier Profilen sinnvoll erscheint. Vergleicht man diese Modelle mit Blick auf Interpretierbarkeit und Theoriebezug, zeigen sich beim Drei-Profil-Modell konsistente Profile mit je hohen, mittleren bzw. tiefen Ausprägungen auf allen Variablen. Im Modell mit vier Profilen sind differenziertere Gruppen auszumachen, die sich im Kontext bereits vorliegender Forschungsergebnisse (z. B. Linnenbrink-Garcia et al. 2012; Seidel 2006) gut interpretieren lassen. Aus diesem Grund wurde eine Entscheidung für das Modell mit vier Profilen getroffen.

**Figure 1:**
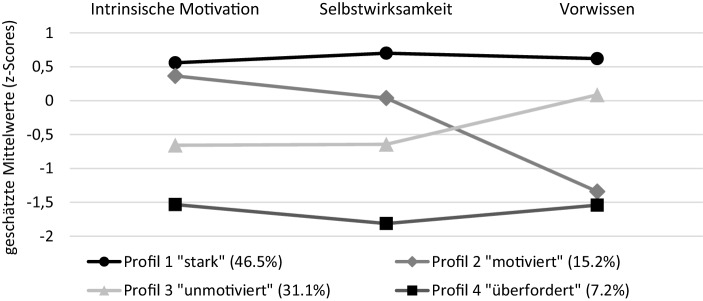

In Abb. 1 sind die Ergebnisse der Vier-Profil-Lösung dargestellt. Entsprechend unserer Annahme H1 konnten sowohl konsistente als auch inkonsistente Merkmalskonfigurationen identifiziert werden. Das konsistente Profil 1 umfasst mit 46,5 % den größten Anteil der Schüler*innen mit hoch ausgeprägter *Selbstwirksamkeit*, hoch ausgeprägter *intrinsischer Motivation* und hoch ausgeprägtem *Vorwissen* in Mathematik (*starkes Profil*). Das zweite konsistente Profil 4 schließt mit 7,2 % eine kleine Gruppe von Lernenden mit niedrigen Werten auf allen drei Variablen ein (*überfordertes Profil*). Bei Profil 2 und Profil 3 zeigen sich Inkonsistenzen zwischen den motivationalen und den kognitiven Voraussetzungen. Profil 2 mit hoch ausgeprägter *intrinsischer Motivation*, durchschnittlicher *Selbstwirksamkeit* und geringem *Vorwissen* in Mathematik (*motiviertes Profil*) umfasst 15,2 % der Lernenden. Profil 3, dem sich 31,1 % der Lernenden zuordnen lassen, ist charakterisiert durch eine geringe *Selbstwirksamkeit und intrinsische Motivation* sowie durch mittlere Werten im *Vorwissen* (*unmotiviertes Profil*). Die vier Schüler*innenprofile unterscheiden sich in den drei untersuchten Indikatoren signifikant voneinander (Overall-Test, *df* = 3;* intrinsische Motivation:* χ^2^ = 460,73; *p* < 0,001; *Selbstwirksamkeit:* χ^2^ = 940,68; *p* < 0,001; *Vorwissen:* χ^2^ = 897,018; *p* < 0,001). Im paarweisen Vergleich zeigen sich lediglich in der *intrinsischen Motivation* zwischen dem motivierten und dem starken Profil keine signifikanten Unterschiede (χ^2^ = 0,12; *p* = 0,74). Gleiches gilt für das *Vorwissen* im motivierten und im überforderten Profil (χ^2^ = 0,00; *p* = 1,00).

Zusätzlich zur Analyse der kognitiv-motivationalen Profile wurde untersucht, ob sich die Voraussetzungen geschlechterspezifisch und für mehrsprachige Schüler*innen unterscheiden. Als Referenzgruppe wurde Profil 1 (starkes Profil) herangezogen, da bei Schüler*innen dieses Profils idealtypische Ausprägungen der Variablen vorliegen. Die Ergebnisse der multinominalen statistischen Regression zeigen signifikante Wahrscheinlichkeitsunterschiede in Abhängigkeit von Geschlecht und Mehrsprachigkeit. Eine Ausnahme bildet das motivierte Profil im Zusammenhang mit dem Geschlecht (vgl. Tab. 3).

**Table 3:** 

	Profil 2 „motiviert“					Profil 3 „unmotiviert“					Profil 4 „überfordert“				
	*b*	b*	*SE*	*OR*	*p*	*b*	b*	*SE*	*OR*	*p*	*b*	b*	*SE*	*OR*	*p*
Geschlecht	0,07	0,04	0,05	1,08	0,15	0,56	0,28	0,07	1,75	0,00	0,34	0,17	0,09	1,41	0
Mehrsprachigkeit	0,59	0,29	0,06	1,80	0,00	−0,39	−0,19	0,07	0,68	0,00	−0,49	−0,24	0,09	0,62	0

*b* nicht standardisierter Regressionskoeffizient, b* partiell standardisierter logistischer Regressionskoeffizient, *SE* Standardfehler, *OR* Odds Ratio, *p* Signifikanzwert

In Tab. 4 werden die konkreten Wahrscheinlichkeiten aufgeführt, die zur besseren Interpretation berechnet wurden. Diese veranschaulichen Abweichungen in den Profilzugehörigkeitswahrscheinlichkeiten für Mädchen und Jungen sowie für Kinder mit und ohne Mehrsprachigkeit.

**Table 4:** 

Prädiktor	Ausprägung	Profil 1 „stark“ (in %)	Profil 2 „motiviert“ (in %)	Profil 3 „unmotiviert“ (in %)	Profil 4 „überfordert“ (in %)
Geschlecht	Mädchen	41,9	13,7	36,9	7,6
	Jungen	52,4	15,9	25,8	6,8
Sprache	Mehrsprachigkeit	48,1	21,9	24,6	5,5
	Keine Mehrsprachigkeit	45,6	11,5	34,5	8,4
Profilwahrscheinlichkeiten Gesamtstichprobe:		46,5	15,2	31,1	7,2

Mädchen sind im starken Profil unterrepräsentiert und haben im Vergleich zur Gesamtstichprobe mit 41,9 % die geringste Wahrscheinlichkeit für dieses Profil. Im unmotivierten Profil sind sie hingegen überdurchschnittlich häufiger vertreten. Jungen haben im Vergleich dazu mit größerer Wahrscheinlichkeit ein starkes und mit geringerer Wahrscheinlichkeit ein unmotiviertes Profil. Mehrsprachige Schüler*innen sind im motivierten Profil deutlich überrepräsentiert und gleichzeitig im unmotivierten Profil sowie im überforderten Profil, die beide durch niedrige motivationale Werte gekennzeichnet sind, unterrepräsentiert.

### Wahrgenommene Lernunterstützung

**Figure 2:**
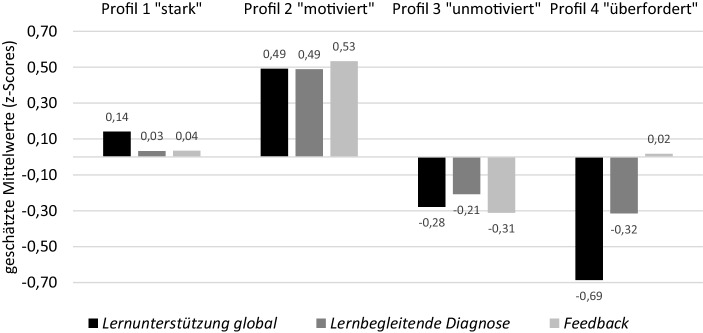

Anhand der zweiten Forschungsfrage wurde untersucht, ob Schüler*innen mit verschiedenen Profilen die Lernunterstützung im Unterricht unterschiedlich wahrnehmen (F2). Konform mit Hypothese H2.1 zeigen die Ergebnisse der multiplen Gruppenvergleiche in der Wahrnehmung der Lernunterstützung insgesamt signifikante Unterschiede zwischen den Profilen, und dies sowohl im Hinblick auf die globale Einschätzung der Lernunterstützung als auch in der Einschätzung der lernbegleitenden Diagnose und des Feedbacks (Overall-Test, *df* = 3;* Lernunterstützung global:* χ^2^ = 34,97; *p* < 0,001; *lernbegleitende Diagnose:* χ^2^ = 21,16; *p* < 0,001; *Feedback:* χ^2^ = 15,152; *p* < 0,001). Teilweise erwartungskonform berichteten Lernende mit einem konsistenten starken Profil leicht positive bis neutrale Wahrnehmungen der drei untersuchten Aspekte von Lernunterstützung. Erwartungswidrig ließ sich demgegenüber nicht für das starke Profil, sondern für Schüler*innen mit einem inkonsistenten motivierten Profil (geringes Vorwissen, aber hoch ausgeprägte Motivation) insgesamt die positivste Wahrnehmung der Lernunterstützung feststellen. Kinder mit einem unmotivierten und überforderten Profil nahmen die Lernunterstützung im Vergleich tendenziell negativ wahr (vgl. Abb. 2). Ebenfalls unerwartet schätzten Schüler*innen mit einem konsistenten überforderten Profil (niedrige Variablenausprägungen) die Lernunterstützung global und die lernbegleitende Diagnose der Lehrperson zwar negativ ein, das Feedback jedoch neutral. Unsere Hypothese H2.2, dass Lernende mit geringen motivationalen Ausprägungen und gleichzeitig hoch ausgeprägtem Vorwissen (unmotiviertes Profil) die Lernunterstützung der Lehrperson negativer wahrnehmen als Schüler*innen mit einem starken Profil, konnte somit bestätigt werden. Insbesondere das Feedback wurde von diesen Schüler*innen negativ eingeschätzt.

Die größten Unterschiede in der Wahrnehmung zeigten sich mit mehr als einer Standardabweichung zwischen der positiven Wahrnehmung der Lernunterstützung global im motivierten und der negativen Einschätzung derselben im überforderten Profil sowie in der Wahrnehmung des Feedbacks zwischen dem motivierten und dem unmotivierten Profil.

Die differenzielle Untersuchung der Schüler*innenwahrnehmung der Komponenten von Lernunterstützung ergab auch im Paarvergleich Unterschiede zwischen den Profilen.

#### Lernunterstützung global

Im paarweisen Vergleich der Profile sind die Unterschiede in den globalen Wahrnehmungen der Lernunterstützung für alle Profile signifikant (χ^2^ = 3,83–29,20; *p* = 0,00–0,05) und eine leicht positivere Wahrnehmung von Kindern mit starkem Profil bestätigte sich. Jedoch zeigten auch hier Schüler*innen mit einem motivierten Profil die positivste Wahrnehmung.

#### Lernbegleitenden Diagnose

In Wahrnehmung der lernbegleitenden Diagnose unterschied sich in der positiven Wahrnehmung nur das motivierte Profil signifikant von den anderen Profilen (motiviert vs. stark: χ^2^ = 6,14; *p* = 0,01; motiviert vs. unmotiviert: χ^2^ = 17,22; *p* = 0,00; motiviert vs. überfordert: χ^2^ = 13,87; *p* = 0,00). In der Tendenz erwiesen sich auch Unterschiede zwischen dem starken und dem unmotivierten Profil (χ^2^ = 2,72; *p* = 0,10) sowie zwischen dem starken und dem überforderten Profil (χ^2^ = 3,46; *p* = 0,06) als signifikant. Zur negativsten Einschätzung gelangten Lernende im überforderten Profil.

#### Feedback

Die Ergebnisse zur Wahrnehmung von Feedback sind nur teilweise erwartungskonform. Schüler*innen mit einem starken Profil schätzten das Feedback neutral bis leicht positiv ein. Jedoch bestand ein signifikanter Unterschied nur zu den stark negativen Einschätzungen der Lernenden mit unmotiviertem Profil (χ^2^ = 6,03; *p* = 0,01). Die Unterschiede in der Wahrnehmung von Feedback zwischen dem starken und dem überforderten Profil fielen entgegen den Annahmen nicht signifikant aus (χ^2^ = 0,01; *p* = 0,92). Wie in Abb. 2 zu erkennen ist, hatten Kinder im überforderten Profil im Hinblick auf das Feedback eine eher neutrale Wahrnehmung. Erwartungskonform hingegen war die Wahrnehmung des Feedbacks beim unmotivierten Profil deutlich und signifikant negativer als beim starken Profil (χ^2^ = 6,03; *p* = 0,01) und beim motivierten Profil (χ^2^ = 6,35; *p* = 0,01).

## Diskussion

Wie Schüler*innen den Unterricht und insbesondere die Lernunterstützung ihrer Lehrpersonen individuell wahrnehmen und beurteilen, ist von hoher Relevanz für ihren Lernprozess (z. B. Harks et al. 2014; Schenke 2018). In Erweiterung zu bestehenden Studien fokussierte die vorliegende Untersuchung auf Erklärungen für diesbezügliche Wahrnehmungsunterschiede mit der Absicht, einen Einblick in die differenzielle Wahrnehmung der Lernunterstützung durch Schüler*innen mit unterschiedlichen kognitiven und affektiv-motivationalen Voraussetzungen zu geben. Zur Analyse der Voraussetzungen wurden Schüler*innenprofile erstellt, da es solche personenzentrierten Auswertungen erlauben, die Komplexität der individuellen Lernvoraussetzungen von Schüler*innen adäquater abzubilden als Analysen einzelner Merkmale, wie sie etwa in variablenzentrierten Ansätzen vorgenommen werden (z. B. Pastor et al. 2007). Erkenntnisse zu Zusammenhängen zwischen den Profilen und der wahrgenommenen Lernunterstützung können dazu beitragen, die heterogenen Bedürfnisse von Schüler*innen hinsichtlich der individuellen Lernunterstützung durch die Lehrperson differenzierter zu verstehen und so Schlüsse für eine adaptive Gestaltung von Lernunterstützung zu ziehen.

### Schüler*innenprofile

Entsprechend unseren Hypothesen konnten entlang der drei Merkmale *intrinsische Motivation, Selbstwirksamkeit* und *Vorwissen* im Fach Mathematik vier Schüler*innenprofile mit sowohl konsistenten (*starkes Profil* und *überfordertes Profil*) als auch inkonsistenten Merkmalskonfigurationen (*motiviertes Profil* und *unmotiviertes Profil*) identifiziert werden. Das konsistente *starke Profil* umfasste mit fast der Hälfte der Lernenden die größte Gruppe. Dies ist aufgrund der günstigen kognitiv-motivationalen Lernvoraussetzungen als gute Grundlage für mathematische Lernprozesse zu bewerten. Erste Längsschnittanalysen zeigen zudem, dass starke Profile über die Zeit recht stabil sind (Lazarides et al. 2018). Lernende mit *überfordertem Profil *und eher ungünstigen, konsistent niedrigen kognitiv-motivationalen Merkmalausprägungen, machten im Gegensatz dazu die kleinste Gruppe aus.

In den vorliegenden Daten für die Primarstufe konnten des Weiteren nicht nur ein (vgl. Südkamp et al. 2018), sondern zwei inkonsistente Schüler*innenprofile identifiziert werden (*motiviertes Profil* und *unmotiviertes Profil*), bei denen das Vorwissen in Mathematik und die motivationalen Variablen* intrinsische Motivation* und *Selbstwirksamkeit* nicht konvergierten. Bislang wurde in den wenigen verfügbaren Studien im Bereich der Primarschule eine geringere Anzahl an Profilen identifiziert (Südkamp et al. 2018), bzw. es ließen sich lediglich Inkonsistenzen zwischen dem Selbstkonzept und der intrinsischen Motivation feststellen (Lazarides et al. 2018). Die vorliegenden Profile gleichen hingegen jenen, die im Bereich der Sekundarstufe gefunden wurden (Linnenbrink-Garcia et al. 2012; Seidel 2006) und deuten darauf hin, dass Schüler*innen bereits in der Primarschule komplexe Merkmalskonfigurationen mitbringen, die einen Einfluss auf das Lernen haben können. Bisher bestand in der Diskussion um personenzentrieren Ansätze wenig Konsens darüber, welche Merkmale die Voraussetzungen von Lernenden im Hinblick auf die Wahrnehmung von Unterricht am aussagekräftigsten abbilden. Die vorliegenden Ergebnisse sprechen dafür, im Gegensatz zur Analyse rein motivationaler Schüler*innenprofile (z. B. Lazarides et al. 2019) sowohl kognitive als auch motivationale Merkmale zu berücksichtigen, da die von uns eruierten Inkonsistenzen zwischen diesen Merkmalen für die Wahrnehmung der Lernunterstützung relevant zu sein scheinen (vgl. Abschn. 5.2). In zukünftigen Studien sollte jedoch auch untersucht werden, ob diesbezüglich noch weitere Variablen von Bedeutung sind, z. B. die Gewissenhaftigkeit oder die Ängstlichkeit.

Die Profilanalyse zeigte des Weiteren, dass Mädchen seltener dem *starken Profil* und öfter dem *unmotivierten Profil* angehörten. Dies ließe sich u. a. anhand gendertypischer Sozialisierungsprozesse erklären, von denen Mädchen in Mathematik schon in der Primarschule oftmals betroffen sind (Cvencek et al. 2011) und die dazu führen können, dass sie über eine geringe Selbstwirksamkeit und weniger intrinsische Motivation in Mathematik berichten als Jungen. Ähnliche gendertypische Profilunterschiede wurden auch im Bereich der Sekundarstufe gefunden (Lazarides et al. 2019; Seidel 2006).

Mehrsprachige Lernende waren im *motivierten Profil* deutlich überrepräsentiert und verfügten über eine hoch ausgeprägte intrinsische Motivation, die sich nicht signifikant von jener im *starken Profil* unterschied. Gleichzeitig hatten sie ein geringes Vorwissen, das sich nicht signifikant von jenem im *überforderten Profil* unterschied. Gemäß Kempert et al. (2016) können die sprachlichen Fähigkeiten in der Unterrichtssprache als eine Ursache für mathematische Leistungsdisparitäten gesehen werden, was eine Erklärung für das niedrige Vorwissen dieser Gruppe sein könnte. Außerdem kann ein Zusammenhang mit dem kulturellen Hintergrund der Lernenden vermutet werden. Entsprechend der Angaben zur Mehrsprachigkeit (vgl. Tab. 5 im Anhang) lassen sich die betreffenden Schüler*innen als kulturell vielfältig beschreiben. Die Ergebnisse können daher zumindest in manchen Fällen als Abbildung des „Aspiration-Achievement-Paradox“ interpretiert werden, dem zufolge bei Lernenden mit Migrationshintergrund trotz geringer Kompetenzen oftmals höhere Bildungsaspirationen vorliegen (Becker und Gresch 2016), welche mit einer höheren Motivation einhergehen (Stanat et al. 2010).

Zusammenfassend kann festgehalten werden, dass die vier identifizierten Profile mit dem Geschlecht und der Mehrsprachigkeit zusammenhängen und auf mögliche Risikokonstellationen hinweisen, welche Lehrpersonen im Hinblick auf eine wirksame Förderung von Lernprozessen im Mathematikunterricht im Auge behalten sollten.

### Wahrnehmungsunterschiede

Anknüpfend an variablenzentrierte Studien weisen die vorliegenden Auswertungen darauf hin, dass die Lernunterstützung von Kindern mit unterschiedlichen Schüler*innenprofilen verschieden wahrgenommen wurde. Diese Ergebnisse können dazu beitragen, die Seite der Wahrnehmung und Nutzung von Unterrichtsangeboten aus Schüler*innensicht differenzierter zu verstehen. In empirischen Untersuchungen wurden bisher mehrheitlich klassenweise aggregierte Urteile untersucht (Lüdtke et al. 2009), in denen individuelle Wahrnehmungsunterschiede als „Rauschen“ herausgerechnet wurden. Gemäß den dargelegten Ergebnissen scheint sich hinter solchen individuellen Wahrnehmungsdivergenzen jedoch eine Systematik abzuzeichnen, die sich auf individuelle Ausgangslagen zurückführen lässt und daher zukünftig stärker zu berücksichtigen und explizit zu modellieren wäre. Mit der vorliegenden Untersuchung liegen erstmals Daten darüber vor, wie die Kombination kognitiver und motivationaler Merkmale mit der Wahrnehmung verschiedener Aspekte der Lernunterstützung zusammenhängen kann.

Im Vergleich der Profile ließ sich die positivste Wahrnehmung der Lernunterstützung beim *motivierten Profil* feststellen. Schüler*innen mit einem *starken Profil* nahmen die Lernunterstützung ihrer Lehrpersonen neutral bis positiv wahr, während sie von Lernenden mit *unmotiviertem Profil* negativ und Lernenden mit *überfordertem Profil* sogar sehr negativ eingeschätzt wurde. Diese Unterschiede zwischen den Profilen deuten darauf hin, dass die intraindividuelle Kombination motivationaler und kognitiver Variablen für die Wahrnehmung der Lernunterstützung entscheidend sein dürfte und machen auf die unterschiedlichen Bedürfnisse dieser Gruppen im Hinblick auf die Lernunterstützung durch die Lehrperson aufmerksam. Trotz eines hoch ausgeprägten Vorwissens in Mathematik schätzten beispielsweise Schüler*innen mit einem unmotivierten Profil die Lernunterstützung wesentlich negativer ein als Schüler*innen mit einem starken oder motivierten Profil mit jeweils hoch ausgeprägter Motivation. Solche Schüler*innen werden in Ergebnissen variablenzentrierter Untersuchungen, die Zusammenhänge zwischen einem hoch ausgeprägten Vorwissen und einer positiven Unterrichtswahrnehmung nachweisen konnten (z. B. Igler et al. 2019), nicht abgebildet. Vielmehr scheint das Vorwissen in Kombination mit motivationalen Merkmalen entscheidend für die Wahrnehmung der Lernunterstützung zu sein.

Dieser Befund ist relevant für die Praxis, da solche Wahrnehmungen von Schüler*innen für Lehrpersonen kontraintuitiv sein könnten. Schüler*innen mit *unmotiviertem Profil* verfügen infolge ihres hoch ausgeprägten Vorwissens über vermeintlich günstige Leistungsvoraussetzungen. Wie in Abschn. 2.3 bereits festgehalten wurde, dürften sie bei Lehrpersonen daher im Hinblick auf den Unterstützungsbedarf weniger im Mittelpunkt stehen (Praetorius und Südkamp 2019). Die negative Wahrnehmung der Lernunterstützung von Schüler*innen in diesem Profil könnte jedoch ganz im Gegenteil darauf hindeuten, dass sie einen erhöhten Unterstützungsbedarf haben, der von Lehrpersonen nicht adressiert wird, weshalb sie von der Lernunterstützung möglicherweise nur eingeschränkt profitieren können. Es ist gut vorstellbar, dass sich Schüler*innen mit einem hoch ausgeprägten Vorwissen und geringer Selbstwirksamkeit sowie geringer intrinsischer Motivation aufgrund der geringen Zuversicht in die eigenen Fähigkeiten bei der Bearbeitung mathematischer Aufgaben unsicherer fühlen als Schüler*innen mit stark ausgeprägten motivationalen Voraussetzungen. Dies betrifft in unserer Stichprobe insbesondere Mädchen, die in diesem Profil überrepräsentiert sind. Es ist daher wichtig, dass Lehrpersonen bei der individuellen Lernunterstützung auch die Motivation ihrer Schüler*innen berücksichtigen.

An diesem Punkt kann in der Förderung angesetzt werden. Indem z. B. Kinder mit *unmotivierten Profilen* frühzeitig identifiziert und unterstützt werden, kann ihre Motivation aufgebaut und einem Abfall der Leistungen entgegengewirkt werden. Längsschnittliche Untersuchungen zeigen, dass die vorangegangene Motivation Effekte auf die nachfolgende Leistung haben kann (Gottfried et al. 2007; Guay et al. 2003). Ein Anstieg der Motivation könnte somit zu einer positiveren Wahrnehmung und infolgedessen auch zu einer besseren Nutzung der Lernunterstützung führen. Gleichzeitig gälte es aber auch die Förderung von Schüler*innen mit *motivierten Profilen* nicht zu vernachlässigen, um ihre Motivation trotz geringen Vorwissens weiter aufrechtzuerhalten, was sich für die Lernentwicklung ebenfalls als positiv erweisen dürfte. Andernfalls besteht gemäß längsschnittlichen Befunden die Gefahr, dass die Motivation dieser Kinder zunehmend abnimmt (Lazarides et al. 2018).

Die differenzierte Analyse der Wahrnehmung der verschiedenen Komponenten von Lernunterstützung (Feedback und lernbegleitende Diagnose) zeigt des Weiteren, dass die Wahrnehmungen zwar einem allgemeinen Trend folgen, der sich auf die kognitiven und die motivationalen Unterschiede zwischen den Profilen zurückführen lässt, dass dieses Muster jedoch beim Feedback durchbrochen wird. So wurde das Feedback der Lehrperson von Schüler*innen mit *starkem* und *überfordertem Profil* neutral bis positiv und somit ähnlich eingeschätzt, obwohl zu erwarten gewesen wäre, dass Schüler*innen mit *überfordertem Profil*, dem Trend folgend, eher eine negativere Wahrnehmung aufweisen würden. Eine Erklärung dafür könnte darin bestehen, dass die Wahrnehmung je nach betrachtetem Unterrichtsaspekt variiert. Zu ähnlichen Ergebnissen gelangten auch Igler et al. (2019). Ihre Untersuchungen zeigen, dass die Einschätzung verschiedener Aspekte der Unterrichtsqualität durch unterschiedliche Schüler*innenmerkmale stärker oder weniger stark vorhergesagt wurde. Folglich müssten einzelne Aspekte der Lernunterstützung wie das Feedback künftig detaillierter in den Blick genommen werden. Zugleich wäre aber auch denkbar, dass überforderte Lernende im Vergleich zu anderen Unterstützungshandlungen häufiger Feedback von der Lehrperson erhalten hatten. Befunde von Lehrpersonenbefragungen deuten in diese Richtung. So gaben Lehrpersonen laut einer Studie von Kiuru et al. (2015) an, mit leistungsschwachen Schüler*innen mehr Zeit im Unterricht zu verbringen. Diesbezüglich gälte es jedoch genau zu eruieren, welchen Umfang das Feedback während der Unterstützung eingenommen hatte.

### Limitationen und Forschungsbedarf

Die in den vorhergehenden beiden Abschnitten vorgenommene Interpretation der Ergebnisse unterliegt gewissen Limitationen, die nachfolgend dargelegt werden. So wurden die Daten der vorliegenden Untersuchung im Querschnitt erhoben, weshalb keine Aussagen über mögliche kausale Zusammenhänge getroffen werden können. Theoretisch dürfte ein interdependenter Zusammenhang zwischen der Ausprägung kognitiv-motivationaler Schüler*innenprofile und der Lernunterstützung bestehen. Unsere Daten bilden Zusammenhänge zum Zeitpunkt der Erhebung ab, können jedoch die Frage nicht klären, ob die Wahrnehmung der Lernunterstützung durch kognitiv-motivationale Merkmalskonfigurationen beeinflusst wurde oder ob auch eine interindividuell unterschiedliche Lernunterstützung vorlag, die gegebenenfalls Einfluss auf die Entwicklung und die Stabilität der Profile gehabt haben könnte. Insbesondere bei inkonsistenten Profilen wäre es aufschlussreich zu untersuchen, ob und wie äußere Einflussfaktoren (z. B. das Lehrpersonenhandeln, das Klassenklima oder auch die Beziehung der Schüler*innen zur Lehrperson) positive Entwicklungen fördern können.

Um den Zusammenhang von Wahrnehmung und im Unterricht konkret stattfindenden Unterstützungshandlungen zu klären, müssten des Weiteren Beobachtungsdaten berücksichtig werden, was in den vorliegenden Analysen nicht der Fall war. Für die Überprüfung der Vermutung beispielsweise, dass Lernende *mit überforderten Profilen* häufiger Feedback von der Lehrperson erhalten, wären Videoanalysen der Lernunterstützung im Unterricht einzubeziehen. Daraus würden sich für die weitere Forschung offene Fragen wie die folgenden ergeben: Sind Unterstützungshandlungen von Lehrpersonen adaptiv und adressieren sie spezifische Voraussetzungen von Lernenden mit unterschiedlichen Profilen? Profitieren Lernende von nicht adaptiver Unterstützung differenziell?

Im Hinblick auf die Zusammensetzung der Profile könnte zudem, wie in Abschn. 4.1 bereits festgehalten, noch weiteren, nicht in diese Studie einbezogenen Variablen (z. B. Intelligenz, Zielorientierung, Gewissenhaftigkeit, Ängstlichkeit) eine Bedeutung für die Wahrnehmung von Lernunterstützung zukommen. Insgesamt wäre es in der personenzentrierten Forschung erforderlich, noch stärker Theorie geleitet zu überprüfen, welche Voraussetzungen es in Profilanalysen im Zusammenhang mit der Unterrichtswahrnehmung zu berücksichtigen gälte. Hinsichtlich der wahrgenommenen Lernunterstützung scheint unseren Daten gemäß die Motivation ein großes Gewicht zu haben, weshalb dieses Merkmal künftig differenzierter einbezogen werden könnte. Zudem müssten Schüler*innenprofile in Folgestudien im Zusammenhang mit demografischen Merkmalen den kulturellen Hintergrund differenzierter beleuchten, indem verschiedene kulturelle Einflussfaktoren, z. B. Geburtsort der Eltern, Staatsangehörigkeit, Ausgangssprache, sowie der sozioökonomische Status einbezogen werden, was in dieser Studie nicht umgesetzt werden konnte.

In diesem Zusammenhang ist auch der fachliche Bezug der Schüler*innenprofile von Relevanz. Es müsste überprüft werden, ob sich der Zusammenhang zwischen individuellen Voraussetzungen und der Wahrnehmung auch in anderen Fächern, wie z. B. Deutsch, zeigt. Unseren Daten gemäß variieren die kognitiv-motivationalen Merkmale (instabile Merkmale) von Schüler*innen in Abhängigkeit von stabilen Merkmalen (z. B. Geschlecht, Mehrsprachigkeit). Aus anderen Studien, die den direkten Zusammenhang zwischen Geschlecht und Wahrnehmung untersuchten (z. B. Corbin et al. 2020; Igler et al. 2019,), gingen hingegen widersprüchliche Ergebnisse hervor. Gemäß Corbin et al. (2020) nahmen Mädchen im 3. und 4. Schuljahr den Unterricht in Bezug auf das Lesen positiver wahr als Jungen, während Jurik et al. (2015) in Mathematik keine Unterschiede in der Wahrnehmung zwischen Mädchen und Jungen finden konnten. Dies könnte auf Unterschiede in den kognitiven und motivationalen Voraussetzungen zwischen Mädchen und Jungen hindeuten, die möglicherweise auch fachspezifisch variieren und direkt wahrnehmungsrelevant sind. Es besteht daher weiterer Forschungsbedarf zur Frage, wie stabile und instabile Voraussetzungen bei der Unterrichtswahrnehmung interagieren.

Abschließend gilt es auch im Hinblick auf die Operationalisierung der Lernunterstützung auf einige Einschränkungen hinzuweisen. Diesbezüglich könnten noch weitere Merkmale von Lernunterstützung wie das Erklären oder das Vormachen von Bedeutung sein (Krammer 2016; Siemon et al. 2018). Ferner konnte bei den relativ jungen Teilnehmenden dieser Studie das Feedback nur mittels einer einfachen Skala erfasst werden. Es müsste künftig stärker zwischen verschiedenen Arten von Feedback unterschieden (Hattie und Timperley 2007) und könnte zwischen kognitiver und emotionaler Lernunterstützung differenziert werden.

### Fazit und Ausblick

Trotz der großen Bedeutung von individuellen Voraussetzungen für die Wahrnehmung und die Nutzung von Unterrichtsangeboten existierten bisher noch kaum Untersuchungen, die Zusammenhänge zwischen Schüler*innenprofilen und der Wahrnehmung von Unterricht untersucht haben (Lazarides et al. 2019; Seidel 2006). Die vorliegende Untersuchung erweitert den bisherigen Forschungsstand, indem erste Erkenntnisse über die individuelle Wahrnehmung von Lernunterstützung gewonnen werden konnten. Im Kern weist die Untersuchung darauf hin, dass Schüler*innen Unterstützungshandlungen von Lehrpersonen im Unterricht vor dem Hintergrund ihrer kognitiv-motivationalen Merkmalskonfigurationen wahrnehmen und diesbezüglich große Unterschiede zwischen einzelnen Lernenden bestehen. Außerdem scheint – womöglich kontraintuitiv für Lehrpersonen – nicht das Vorwissen als kognitives Merkmal primär für die Wahrnehmung ausschlaggebend zu sein, sondern die individuelle Kombination des Vorwissens mit motivationalen Variablen. Der bisherige Fokus der Forschung zu Zusammenhängen von adaptiver Lernunterstützung und Leistungsunterschieden zwischen Schüler*innen (Lazarides et al. 2018, S. 253) ist aus unserer Sicht deshalb limitiert und müsste durch einen stärkeren Einbezug motivationaler Merkmalen erweitert werden.

Für die Praxis lässt sich daraus ableiten, dass bei der Planung und Durchführung von Lernunterstützung nicht nur auf die Leistungsfähigkeit von Schüler*innen geachtet werden sollte, sondern auch die motivationalen Ausgangslagen in Kombination mit Leistungsvoraussetzungen einbezogen werden müssten, damit individuelle Bedürfnisse adaptiv adressiert werden können. Befunde zur Fähigkeit von Lehrpersonen, individuelle Ausgangslagen von Schüler*innen zu erkennen, weisen jedoch darauf hin, dass es Lehrpersonen schwerfällt, insbesondere Schüler*innen mit inkonsistenten Profilen zu identifizieren (Südkamp et al. 2018). Hier könnten Maßnahmen der Lehrpersonenaus- und -fortbildung ansetzen und ein Wissen über bzw. ein Bewusstsein für mögliche Inkonsistenzen vermitteln. In diesem Zusammenhang gälte es dafür zu sensibilisieren, dass Unterstützungshandlungen von Lernenden unterschiedlich wahrgenommen werden können. Um Lehrpersonen gezielt darüber zu informieren, wie Lernende entsprechend ihren individuellen Merkmalskonfigurationen bestmöglich adaptiv gefördert werden können, ist jedoch zuerst weitere vertiefende Forschung zu Schüler*innenprofilen und der Passung von wahrgenommener und beobachteter Lernunterstützung im Unterricht erforderlich.

## Anhang

**Table 5:**

	Sprache	Prozent
Landessprachen (Schweiz)	Italienisch	3,5
	Französisch	1,1
	Rätoromanisch	0,5
Andere Sprachen	Albanisch	8,0
	Portugiesisch	4,4
	Spanisch	2,8
	Tamilisch	2,1
	Englisch	1,7
	Serbisch	1,7
	Russisch	1,4
	Türkisch	0,9
	Bosnisch	0,8
	Arabisch	0,6
	Chinesisch	0,6
	Finnisch	0,6
	Kroatisch	0,6
	Mazedonisch	0,6
	Rumänisch	0,6
	Tigrinya	0,6
	Niederländisch	0,5
	Kurdisch	0,5
	Griechisch	0,3
	Persisch	0,3
	Philippinisch	0,3
	Polnisch	0,3
	Bulgarisch	0,2
	Dänisch	0,2
	Kantonesisch	0,2
	Schwedisch	0,2
	Slowakisch	0,2
	Tibetisch	0,2
	Tschetschenisch	0,2
	Ungarisch	0,2
	Urdu	0,2
	Valenzianisch	0,2
	Sprache nicht zuordenbar	0,3
–	–	100

## Footnotes

1 Ziel der *TUFA*-Studie ist, das formative Assessment von Lehrpersonen im Unterricht zu analysieren, Prädiktoren zu untersuchen sowie Zusammenhänge mit Schüler*innenvariablen, z. B. der Wahrnehmung, und Lernfortschritten zu beleuchten.
